# Applying EASTL ethical constructs in AI-driven CRM: the mediating role of sustainable customer trust in enhancing customer retention in private sector banks

**DOI:** 10.3389/frai.2026.1865410

**Published:** 2026-08-03

**Authors:** A. Geetha, V. Venkatragavan

**Affiliations:** 1 Department of Commerce, Faculty of Science and Humanities, SRM Institute of Science and Technology, Vadapalani Campus, Chennai, Tamil Nadu, India; 2 Department of Commerce (A&F), Faculty of Science and Humanities, SRM Institute of Science and Technology, Vadapalani Campus, Chennai, Tamil Nadu, India

**Keywords:** artificial intelligence ethics, customer relationship management, customer retention, EASTL theory, ethical AI, private sector banks, sustainable trust

## Abstract

The rapid integration of Artificial Intelligence (AI) in banking, Customer Relationship Management (CRM) has undergone several changes, which have also led to ethical concerns and implications on customer outcomes. While previous studies have studied AI adoption and trust independently, little attention has been placed on how ethical AI dimensions interact and affect customer retention via trust mechanisms. In this sense the objective of our study is to investigate ethical AI practices (using the EASTL framework) and its impact on Customer Retention (CR), with Sustainable Customer Trust (SCT) as mediator factor in private sector banks. A quantitative research design was taken by implementing structured questionnaires for 361 respondents. The model consists of five major ethical AI constructs-Normative Ethical Alignment, Institutional Accountability Mechanisms, Algorithmic Transparency and Explainability, Data Security and Privacy, and Regulatory and Social Legitimacy. The data were processed by Partial Least Squares Structural Equation Modeling (PLS-SEM) to calculate the direct and indirect relationships. The model accounts for 48.4% for Variance in Sustainable Customer Trust and 32.1% for Customer Retention. Algorithmic Transparency, Data Security, Institutional Accountability and Regulatory Legitimacy are the major factors that directly and indirectly affect customer retention via trust suggesting partial mediation. But Normative Ethical Alignment was not significant. Sustainable Customer Trust formed a vital mediating factor of ethical AI practices on the customer retention and its influence from the one-sided effect. The research shows that ethical AI is not only a compliance obligation, but a strategic lever for building trust and long-term customer relationships. These results present useful insights for banks and policymakers into how to develop open and trustworthy AI systems that are responsible and reliable to instill customer trust and sustainability within digital banking environments.

## 1 Introduction

The rapid success of Artificial Intelligence (AI) has dramatically changed Customer Relationship Management (CRM) in the banking industry, including especially at banks and private sector institutions. AI-based technologies, including predictive analytics, chatbots, and personalized recommendation systems have allowed banks to increase operational effectiveness and provide personalization, services, and, more importantly, customers to enhance customer experience. According to prior research, implementation of AI in CRM enhances customer engagement, satisfaction, and loyalty through data-driven decision-making and real-time provisioning of services (Ashrafuzzaman et al., 2025; Shivani et al., 2025; Yousaf et al., 2025). Notwithstanding the potential positive changes, a heavy dependency on AI has raised pressing ethical concerns about data privacy, transparency, accountability, and algorithmic bias and can also reduce customer trust in AI systems (Li, 2024; Yu et al., 2025). It is well-known in the banking industry to be one of the basic factors in customer trust as it impacts long-term customer relationships and retention. Despite offering greater personalized service quality, its effectiveness depends on how consumers perceive these AI solutions to be ethical, secure, and transparent. Previous research stresses that ethical deficiencies in AI systems—such as difficulty of explanation and perceived unfairness—may result in distrust, which would in turn lead to deterioration in customer retention and loyalty (Oyekunle et al., 2024; Mooghali et al., 2024). On the other hand, the use of ethical AI practices has proven to enhance customer confidence and build long-term relationships (Nadeem, 2025; Selvam, 2025). But much of the research so far has not been unified; it’s often siloed down to individual ethical dimensions rather than taking up a holistic set of ethics. The current study responds to this limitation using the EASTL framework, which incorporates Ethics, Accountability, Security, Transparency, and Legitimacy, providing a more inclusive perspective on ethical AI in CRM systems. Together, these dimensions capture the technical and governance-related aspects of AI adoption, ensuring a more holistic focus on ethical implications. In this regard, five independent factors were utilized in this study, ethics, accountability, transparency, security, and legitimacy to investigate their impact on consumer trust and retention within private sector banks. Adding these dimensions to a model, the study seeks to contribute to the literature by developing a more holistic view into ethical AI in banking.

This research finds that every one of the different EASTL dimensions positively affects customer trust and therefore improves customer retention. Additionally, to mediate the relationship, we expect customer trust as a mediator between ethical AI practices and retention outcomes. We are suggesting that the following hypothesis is based on relationship marketing theory, where trust is an essential precursor of customer loyalty and long-term engagement (Nwachukwu and Chima, 2025). The importance of the findings and implications of this work both in theory and practical terms remains. It advances the literature by the theoretical method of the integration of the principles of ethical AI together with CRM and customer relationship outcomes in a well-structured study. On a practical basis, it is a practical guide for banks on how to build responsible AI systems that engender customer trust and consumer stickiness. Moreover, the investigation also tackles new issues in the area of digitalization and ethical governance as these concerns are related to digital transformation, and its significance and implications of this in the field of private sector banking. The rapid deployment of AI-driven CRM systems in banking has transformed customer interactions through personalized recommendations, automated service delivery, and predictive analytics. However, increasing dependence on AI has simultaneously generated concerns regarding algorithmic transparency, accountability, fairness, privacy protection, and regulatory compliance. These concerns have become particularly significant as customers increasingly rely on AI-mediated financial services while possessing limited visibility into how AI systems make decisions. Consequently, understanding how ethical AI practices influence customer trust and retention has become both a theoretical and managerial priority. Given the growing emphasis on responsible AI governance in financial services, this study provides timely evidence regarding the role of ethical AI dimensions in fostering sustainable customer trust and long-term customer retention. Existing studies have investigated individual dimensions of ethical AI and customer trust in digital service environments. For example, Li (2024) examined transparency and privacy concerns associated with AI implementation, while Yu et al. (2025) focused on the relationship between ethical perceptions and AI adoption. Similarly, Mooghali and Stroud (2024) explored privacy and security challenges in trustworthy AI systems, whereas Nadeem (2025) investigated ethical AI-driven customer engagement. Although these studies provide valuable insights, they primarily examine isolated ethical dimensions rather than integrating multiple ethical governance factors into a unified framework. Furthermore, prior research has rarely investigated how ethical AI practices collectively influence customer retention through sustainable customer trust in AI-enabled CRM environments. Consequently, limited empirical evidence exists regarding the mechanisms through which ethical AI governance contributes to long-term customer relationship outcomes in banking. This study addresses this gap by applying the EASTL framework and examining sustainable customer trust as a mediating mechanism between ethical AI practices and customer retention.

*RQ*: *How do EASTL-based ethical AI practices in CRM affect customer trust and subsequently customer retention in private sector banks?*

This study adopted five independent factors—normative ethical alignment, institutional accountability mechanisms, algorithmic transparency and explainability, data security and privacy resilience, and regulatory and social legitimacy—derived from the Ethical AI-Driven Sustainable Trust Loop (EASTL) Theory to examine their influence on sustainable customer trust, which serves as a mediating factor in AI-driven Customer Relationship Management (CRM) systems within private sector banks. These dimensions make up the general pillars of ethical artificial intelligence and touch on such important questions as the principles of fairness, responsibility, data protection, and regulatory compliance. Previous studies show that users are more willing to trust in AI models which are driven by ethical factors including transparency and accountability, while issues related to privacy and security can undermine trust in AI systems if not adequately handled (Li, 2024; Ogeawuchi et al., 2024; Yu et al., 2025). Combining these five factors in an overall framework, the final study seeks to offer insights into how ethical AI practices influence customers’ perceptions and trust which ultimately affect long-term customer retention.

The paper is structured as follows: it begins with the theoretical foundation and conceptual framework, followed by the methodology and data collection approach. Subsequently, the results are analyzed, practical recommendations are presented, and finally, the study concludes with a discussion of implications and contributions.

## 2 Literature review

This study contributes to the literature in three important ways. First, it extends ethical AI research by empirically validating the EASTL framework within AI-driven CRM systems in the banking sector. Second, it advances relationship marketing and trust theory by examining sustainable customer trust as a mediating mechanism linking ethical AI practices to customer retention. Third, it provides practical evidence for banking managers and policymakers regarding which ethical AI dimensions most effectively promote customer trust and long-term customer retention in AI-enabled banking environments.

### 2.1 Theoretical background and study hypotheses

**Figure 1:**
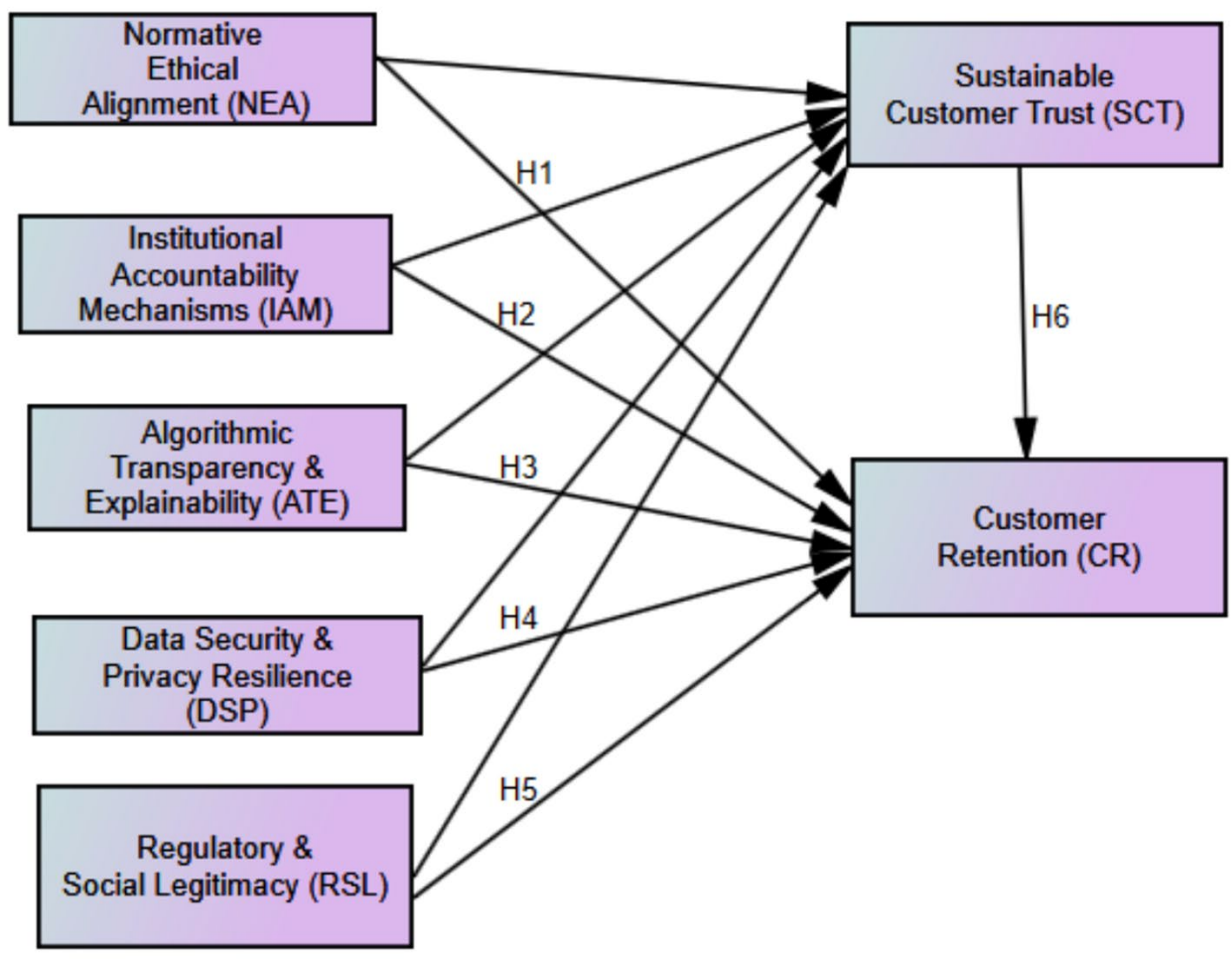
Conceptual framework structural model including direct and indirect paths for mediation analysis.

The Ethical AI-Driven Sustainable Trust Loop (EASTL) model is developed on a multi-theoretical basis, integrating Trust Theory (TT), Stakeholder Theory (ST), and Institutional Theory (IT) on how its ethical AI practices affect customer decision processes in AI-driven CRM systems. TT forms the central explanation by establishing trust as a mediating variable that determines customer retention (Morgan and Hunt, 1994). By contrast, ST also focuses more on the ethical mandate on organizations to align AI systems with stakeholder expectations, especially with regard to customers (Freeman, 1984). IT clarifies how legitimacy and trust are affected by organizational structures, governance mechanisms, and regulatory compliance (Scott, 2001). As shown in Figure 1, the five ethical dimensions — normative ethical alignment, institutional accountability mechanisms, algorithmic transparency and explainability, data security and privacy resilience, and regulatory and social legitimacy — are treated as antecedents for sustainable customer trust that eventually informs customer retention within this framework accountability and ethical (Alshurideh et al., 2024).

#### 2.1.1 Normative ethical alignment

Normative Ethical Alignment (NEA) is a term used to describe the match or ‘Alignment’ of AI systems with ethical principles such as fairness, justice, and non-discrimination. Mullangi et al. (2018) state that, if we take the customer value perspective for which the AI system is in line the organization can build on trust then we can have a fair and mutual cooperation between organisations and the customers. Also, Nadeem (2025) underscores the turn toward authenticity-based AI-models adopting ethical protections. Additionally, Marilahimbilu Mgiba and Mxotwa (2024) establish that clear communicating of ethical AI practices eliminates customer concerns related to bias and discrimination, while Elliott et al. (2021) highlight the importance of nurturing a digital ethics culture in companies. Russo et al. (2023) argue ethical alignments must be embedded in system design to address epistemic asymmetries. Kannan (2024a,b) emphasizes need to reduce algorithmic biases from historical data. Kurshan et al. (2021) list “fairness and non-discrimination” as an international ethical principle for AI systems. From a theoretical point of view, NEA is underpinned by the Stakeholder Theory (ST), which stipulates that organizations should develop AI, including software systems that are designed to protect the interests of stakeholders and to work within and for fairness (Freeman, 1984). Further Trust Theory (TT) also says that people perceive fairness as an input into AI systems as an instrument of trust (Morgan and Hunt, 1994). Hence, ethical alignment reduces perceived injustice and builds trust networks.

*H1: Normative Ethical Alignment has a significant positive effect on Customer Retention.*

#### 2.1.2 Institutional accountability mechanisms

Institutional Accountability Mechanisms (IAM) refer to the governance structures and oversight processes that ensure responsible AI usage. Türkşen et al. (2024) highlight the importance of traceability in enabling organizations to audit AI decisions. Tavasoli et al. (2025) emphasize the need for clearly defined human accountability structures, while Percy et al. (2021) propose a “three-line of defense” model involving compliance, audit, and external oversight. Russo et al. (2023) argue that accountability mechanisms should empower users to question AI decisions, and Fundira and Mbohwa (2025) advocate for comprehensive ethical governance frameworks. Kannan (2024a,b) highlights the need for continuous upskilling to manage AI responsibly, while Krijger (2023) emphasizes interdisciplinary expertise in ethical AI implementation. Theoretically, IAM is strongly grounded in Institutional Theory (IT), which explains how formal structures and governance mechanisms ensure organizational legitimacy (Scott, 2001). Additionally, Trust Theory (TT) supports IAM, as accountability reduces uncertainty and enhances trust in organizational systems (Morgan and Hunt, 1994).

*H2: Institutional Accountability Mechanisms has a significant positive effect on Customer Retention.*

#### 2.1.3 Algorithmic transparency and explainability

Algorithmic Transparency and Explainability (ATE) is the degree to which AI decision-making processes can be understood by users. Berezin (2025) also shows the importance of interpretability to customer trust and loyalty. Ni (2024) finds that perceived innovation and accuracy are two critical factors behind trustworthiness in explainable systems. One issue Ayadurai and Joneidy (2021) highlight is that customers need to know decisions such as loan approvals to prevent dissatisfaction. A gap in explainability research is noted by Kanaparthi (2024), while Kannan (2024a,b) highlights the role of explainability in bias detection. Valavan (2023) offers empirical evidence connecting a lack of transparency with customer dissatisfaction. As per Trust Theory (TT), ATE is backed by the reduction of information asymmetry that affects the perceived reliability (Morgan and Hunt, 1994). This is also reflected in Institutional Theory (IT), as transparency is a requirement of regulatory compliance and of organizational legitimacy (Scott, 2001).

*H3: Algorithmic Transparency & Explainability has a significant positive effect on Customer Retention.*

#### 2.1.4 Data security and privacy resilience

Data Security and Privacy Resilience (DSP) refers to protecting customer data and ensuring compliance with privacy regulations. Kumar et al. (2025) highlight the value of privacy-by-design practices, and Mullangi et al. (2018) emphasize the importance of ethical data protection as an essential condition for trust. Marilahimbilu Mgiba and Mxotwa (2024) observe that communicating security features enhances customer satisfaction. When trust is built, customers are more likely to disclose data. According to Kanaparthi (2024), customers are more willing to share data when trust is established, while Kannan (2024a,b)advises the use of privacy-preserving technologies, such as differential privacy. Oyeniyi et al. (2024) highlight the importance of adapting to changing regulations, and Munira, (2025a,b) show advancements in financial security through AI-driven fraud detection. Theoretically, DSP is based on Stakeholder Theory (ST) as it highlights the organizations have a duty to protect customer data and provide ethical use of data (Freeman, 1984). It is further substantiated by Trust Theory (TT): robust privacy protections lower perceived risk and improve trust (Morgan and Hunt, 1994).

*H4: Data Security & Privacy Resilience has a significant positive effect on Customer Retention.*

#### 2.1.5 Regulatory and social legitimacy

Regulatory and Social Legitimacy (RSL) describes compliance with laws and conforming to societal expectations. Yoganathan et al. (2025) demonstrate that regulatory compliance reduces AI-related concerns and increases trust. Torkestani and Mansouri (2025) emphasize that compliance guarantees transparency and accountability, whereas Orji (2024a, 2024b) reinforces inclusiveness and dynamic compliance frameworks. Adeyelu et al. (2024) call for proactive regulatory strategies, and Türkşen et al. (2024) point to reputational risks as factors of compliance. Kannan (2024a, 2024b) presents “compliance by design,” and Fundira and Mbohwa (2025) emphasize the importance of harmonized regulations. In theory, RSL is well supported by Institutional Theory (IT), which suggests that compliance with rules and norms creates higher degrees of legitimacy (Scott, 2001). Furthermore, Trust Theory (TT) posits that perceived legitimacy helps in reducing uncertainty and increasing trust (Morgan and Hunt, 1994).

*H5: Regulatory & Social Legitimacy has a significant positive effect on Customer Retention.*

#### 2.1.6 Sustainable customer trust (mediator)

Sustainable Customer Trust (SCT) is a long-term and stable belief in the reliability, integrity, and ethical functioning of AI-driven Customer Relationship Management (CRM) systems. In more automated digital banking systems, trust stands at the forefront as an important factor for customer adoption and engagement. Previous studies suggest that trust diminishes perceived risk and uncertainty, especially in technology-mediated services where clients lack direct control over decision-making processes (Gefen et al., 2003). Likewise, as Morgan and Hunt's (1994) commitment-trust theory describes, trust is a key factor that impacts long-term relational exchanges—thus its centrality in CRM frameworks. In AI-informed banking systems, trust is formed as a function of several factors including reliability, transparency, and data privacy. Customers are likely to trust AI systems that show steady performance and ethical behavior according to research (Huang and Rust, 2020). Additionally, the explainability and fairness of algorithms also influence trust and are responsible for skepticism and resistance to opaque algorithms. Studies additionally report a “trust gap” between the customers of digital finance and the companies providing the services and explain why they are skeptical of these AI-driven services based on their concerns regarding bias, data misuse, and lack of accountability. Responsible AI practices with transparency, fairness, and privacy protection have been shown to achieve much higher levels of trust through mitigating such challenges. Organizations that embrace responsible AI frameworks and communicate their ethical commitments to customers effectively tend to engender higher customer trust, for example. Moreover, personalized and responsive AI-driven interactions can also enhance confidence, by enhancing customer experience and perceived value. But keeping the trust takes a process of iterative reinforcement, which often requires steady ethical practice and regulatory compliance. In the EASTL framework, SCT is the principal mediating construct connecting ethical AI dimensions to behavioral outcomes. It reflects not only initial trust building but also trust-raising over time through interactions. That is why maintaining the trustworthiness and trust of customers long-term remains critical to the long-term involvement and loyalty in the AI-led CRMs; sustainable customer trust is vital.

*H6: Sustainable Customer Trust mediates the relationship between EASTL Ethical Constructs and Customer Retention.*

## 3 Methodology

### 3.1 Population and sample

The study utilizes a quantitative and explanatory research design to investigate how Ethical AI-driven constructs that use the EASTL framework contribute to customer retention in AI-enabled banking services, particularly the mediating role of sustainable customer trust. The study adopts a deductive research paradigm, grounded in established literature on customer relationship management, ethical AI, and trust formation, with a specific focus on minor inductive refinement in response to initial qualitative feedback.

The target population consists of bank customers who actively use AI-driven banking services in areas such as digital banking platforms, automated customer support, and AI-based financial recommendations. This group constitutes the most relevant section for assessing perceptions of ethical AI practices and their effects on both trust and retention. A pilot study was initially conducted with 30 respondents to assess the clarity, reliability, and contextual relevance of the questionnaire. Minor changes were made to the wording and comprehension of items on algorithmic transparency and ethical alignment according to feedback. The pilot results confirmed satisfactory internal consistency and content validity.

For the main study, data were collected using a structured questionnaire administered through a purposive sampling technique, targeting respondents with prior experience in AI-enabled banking services. The final sample consisted of 361 respondents, which exceeds the recommended minimum threshold for Partial Least Squares Structural Equation Modeling (PLS-SEM), particularly for models involving multiple constructs and mediation analysis. PLS-SEM was selected due to its suitability for complex predictive models, non-normal data distributions, and relatively large construct structures. The data analysis procedure includes evaluation of the measurement model through reliability and validity assessments, followed by structural model analysis to test the hypothesized relationships. Model evaluation is supported by key indicators such as path coefficients, coefficient of determination (R^2^), predictive relevance (Q^2^), and model fit indices such as the Standardized Root Mean Square Residual (SRMR). This approach facilitates rigorous empirical testing and strengthens the validity of the proposed conceptual framework.

### 3.2 Instruments

The items used to measure the latent constructs in this study were adapted from previously validated scales and modified to fit the context of ethical AI-driven customer relationship management (CRM) systems in the banking sector, as presented in Table 1. Specifically, the constructs of Normative Ethical Alignment, Institutional Accountability Mechanisms, Algorithmic Transparency and Explainability, Data Security and Privacy Resilience, Regulatory and Social Legitimacy, Sustainable Customer Trust, and Customer Retention were developed by reviewing relevant literature and adapting items from prior empirical studies. To ensure contextual relevance, wording adjustments were made so that the statements reflected customers’ perceptions of ethical AI practices in AI-enabled banking services.

**Table 1:** Study measures.

Constructs	Details	Sample items
Normative ethical alignment (NEA)	Adapted from Mullangi et al. (2018), Nadeem (2025), and Kannan (2024a,b), 4 items	*The bank’s AI systems reflect ethical principles that align with customer values.*
Institutional accountability mechanisms (IAM)	Adapted from Percy et al. (2021), Tavasoli et al. (2025), 5 items	*The bank has clear accountability systems for AI-driven decisions.*
Algorithmic transparency and explainability (ATE)	Adapted from Lee and Cha (2025), Ayadurai and Joneidy (2021), 5 items	*The bank explains AI-based decisions in a way customers can understand.*
Data security and privacy resilience (DSP)	Adapted from Kumar et al. (2025), Munira, (2025a,b), 4 items	*The bank’s AI systems adequately protect my personal information.*
Regulatory and social legitimacy (RSL)	Adapted from Yoganathan et al. (2025), Adeyelu et al. (2024), 3 items	*The bank’s AI practices comply with relevant legal and ethical standards.*
Sustainable customer trust (SCT)	Adapted from Ni (2024), Nadeem (2025), 5 items	*I trust the bank’s AI-enabled services to consistently act in my best interest.*
Customer retention (CR)	Adapted from Munira, (2025a,b), Ashrafuzzaman et al. (2025), 4 items	*I intend to continue using this bank because of its trustworthy AI services.*

To improve the face validity, content adequacy, and clarity of the instrument, the preliminary questionnaire was evaluated by a panel of academic experts in the fields of artificial intelligence, customer relationship management, and banking services Based on their suggestions, minor revisions were made to improve wording clarity, eliminate ambiguity, and ensure alignment with the objectives of the study. The final questionnaire instrument consisted of two sections. The first section captured respondents’ demographic information, including gender, age, educational qualification, annual income, and frequency of usage of AI-enabled banking services. The second section contained measurement items related to the study constructs: Normative Ethical Alignment (NEA), Institutional Accountability Mechanisms (IAM), Algorithmic Transparency and Explainability (ATE), Data Security and Privacy Resilience (DSP), Regulatory and Social Legitimacy (RSL), Sustainable Customer Trust (SCT), and Customer Retention (CR).

All the items were scored using a 5-point Likert scale, with 5 indicating ‘strongly agree’ and 1 ‘strongly disagree’. This scaling approach was chosen because it is widely used in behavioral and management research to capture respondents’ perceptions and attitudes effectively.

### 3.3 Data collection

A total of 361 structured questionnaires were then surveyed for use of AI-enabled banking services with customers. The survey instrument was given to the respondents (previous users of digital banking, whether AI-based, chatbots, automated recommendations, intelligent banking services, or intelligent banking assistance systems). These questionnaires were accompanied by cover letters explaining the research aims, instructions to ask and answering questions, a statement describing the procedure of the research, and an assurance of confidentiality. The remaining questionnaires were screened for completeness, and only valid responses were retained for analysis. As such, 361 responses were selected for statistical analysis.

According to the demographic profile Table 2 of the respondents, 58% of them were female respondents, and 42% males: it reveals that women engaged far more in AI-enabled banking services than men did. In terms of age, most of the respondents were in the range of 26–35 years (49%) followed by 18–25 years (20%) and respondents aged 46 years and above represented just 12%, indicating that AI-enabled banking services cater primarily to younger and middle-aged customers. Participants with undergraduate degree holders were the most frequent group of respondents (61%), followed by post-graduates (36%), while only 2% of respondents with doctoral qualifications, pointing toward the fact that the AI based banking service is popular among customers who have moderate to high education. For annual income, most respondents were in the ₹3–6 lakhs income bracket (48%), and less than ₹3 lakhs (20%), confirming that AI-enabled banking services are popular for customers in the middle-income level. Likewise, for banking experience, 48% of respondents had 4–7 years of banking experience, and 20% 1–3 years, showing that moderate banking experience customers become more familiar with AI-enabled banking platforms. On the behavioral dimension, 78% were familiar with AI-enabled banking services which indicates high awareness regarding the adoption and implementation of AI in banking processes. Similarly, 77% of our respondents indicated that they have experience with AI-based banking and the majority of surveyed mentioned using Artificial Intelligence customer service tools in chatbots and automated recommendations. As regard to how often they interact with the bank, most of the respondents indicated that they do so “Sometimes” (38%), followed by “Often” (30%), which means customers have regular interaction with banking operations powered by AI. In security perception, 71% of the respondents thought the AI based banking services are secure, reflecting a perception of overall positive for data protection and privacy in AI-based banking systems. Regarding trust-building factors, Data Security was ranked as most important (27%), in fact followed by Transparency (24%), indicating that secure data processing and communication of AI operations are crucial to enhance confidence of customers. 80% of respondents replied that they would use AI-enabled banking services in the future and agreed to them with a heavy attitude toward using AI-driven banking systems in the future. Regarding the impact of ethical AI on trust, 35% stated that ethical AI has a significant influence on trust and 25% said it is very significant, indicating that ethical AI has a major part to play in building trust. Lastly, among the two confidence-building factors as they pertain to the consumer confidence among the customers, Secure Transactions was the most mentioned as a decisive factor (28%), and Clear Explanations was the second (22%) such that customers value secure and clear how AI-enabled banking system works. The empirical results suggest that the AI-driven banking services are predominantly utilized by younger, highly educated, middle-income customers, and that data security, ethical AI practices, and secure transaction behavior are the primary factors in terms of sustainable customer trust and sustainable use intention.

**Table 2:** Respondent’s demographic profile.

Category	Frequency (*n*)	Percent (*n* = 361)
Gender	Male	42
	Female	58
Age	18–25	20
	26–35	49
	36–45	19
	46 and above	12
Educational Qualification	Undergraduate	61
	Postgraduate	36
	Doctorate (Ph. D.)	2
Annual Income	Below ₹3 Lakhs	20
	₹3–6 Lakhs	48
	₹6–10 Lakhs	19
	Above ₹10 Lakhs	14
Years of Banking Experience	1–3 years	20
	4–7 years	48
	8–10 years	19
	Above 10 years	14
Awareness of AI-enabled banking services	Yes	78
	No	22
Usage of AI-based banking services	Yes	77
	No	23
Frequency of interaction	Never	5
	Rarely	13
	Sometimes	38
	Often	30
	Very often	14
Perception of data security	Yes	71
	No	29
Trust factors	Data security	27
	Transparency	24
	Ethical practices	20
	Regulatory compliance	12
	Human support	18
Continued usage intention	Yes	80
	No	20
Ethical AI influence on trust	Not at all	4
	Slightly	11
	Moderately	24
	Significantly	35
	Very significantly	25
Confidence-building factors	Clear explanations	22
	Secure transactions	28
	Human oversight	20
	Regulatory compliance	12
	Fair treatment	18

## 4 Results

### 4.1 Measurement model

The measurement model assesses the reliability and validity of the constructs with their corresponding items. Acceptance of the model is dependent on three criteria: its internal consistency reliability, its convergent validity, and its discriminant validity (Ferine et al., 2021).

Internal consistency reliability, a form of reliability, examines the consistency between the observed variables in a test. Cronbach’s alpha and composite reliability are the traditional criteria for determining this; nevertheless, composite reliability is preferable because it prioritizes the indicators based on their individual reliability (Hair et al., 2019). Composite reliability and Cronbach’s alpha values range from 0 to 1, and both must exceed the 0.6 thresholds to be considered acceptable (Hair et al., 2019; Ferine et al., 2021). As shown in Table 3, the value of both composite reliability and Cronbach’s alpha surpasses 0.6, thus demonstrating internal consistency reliability.

**Table 3:** Convergent validity.

Constructs	Items	Standardized loadings	Composite reliability (CR)	Cronbach’s alpha	AVE
Normative ethical alignment (NEA)	NEA1	0.867	0.911	0.911	0.700
	NEA2	0.754			
	NEA3	0.747			
	NEA4	0.887			
	NEA5	0.914			
Institutional accountability mechanisms (IAM)	IAM1	0.808	0.781	0.802	0.695
	IAM3	0.895			
	IAM4	0.795			
Algorithmic transparency & explainability (ATE)	ATE3	0.878	0.797	0.796	0.711
	ATE4	0.849			
	ATE5	0.801			
Data security & privacy resilience (DSP)	DSP2	0.749	0.839	0.860	0.671
	DSP3	0.882			
	DSP4	0.818			
	DSP5	0.821			
Regulatory & social legitimacy (RSL)	RSL1	0.797	0.862	0.868	0.646
	RSL2	0.852			
	RSL3	0.842			
	RSL4	0.754			
	RSL5	0.767			
Sustainable customer trust (SCT)	SCT1	0.778	0.871	0.880	0.660
	SCT2	0.814			
	SCT3	0.844			
	SCT4	0.749			
	SCT5	0.871			
Customer retention (CR)	CR1	0.797	0.896	0.938	0.701
	CR2	0.935			
	CR3	0.817			
	CR4	0.899			
	CR5	0.721			

IAM2, IAM5, ATE1, ATE2, DSP1 were deleted.

**Figure 2:**
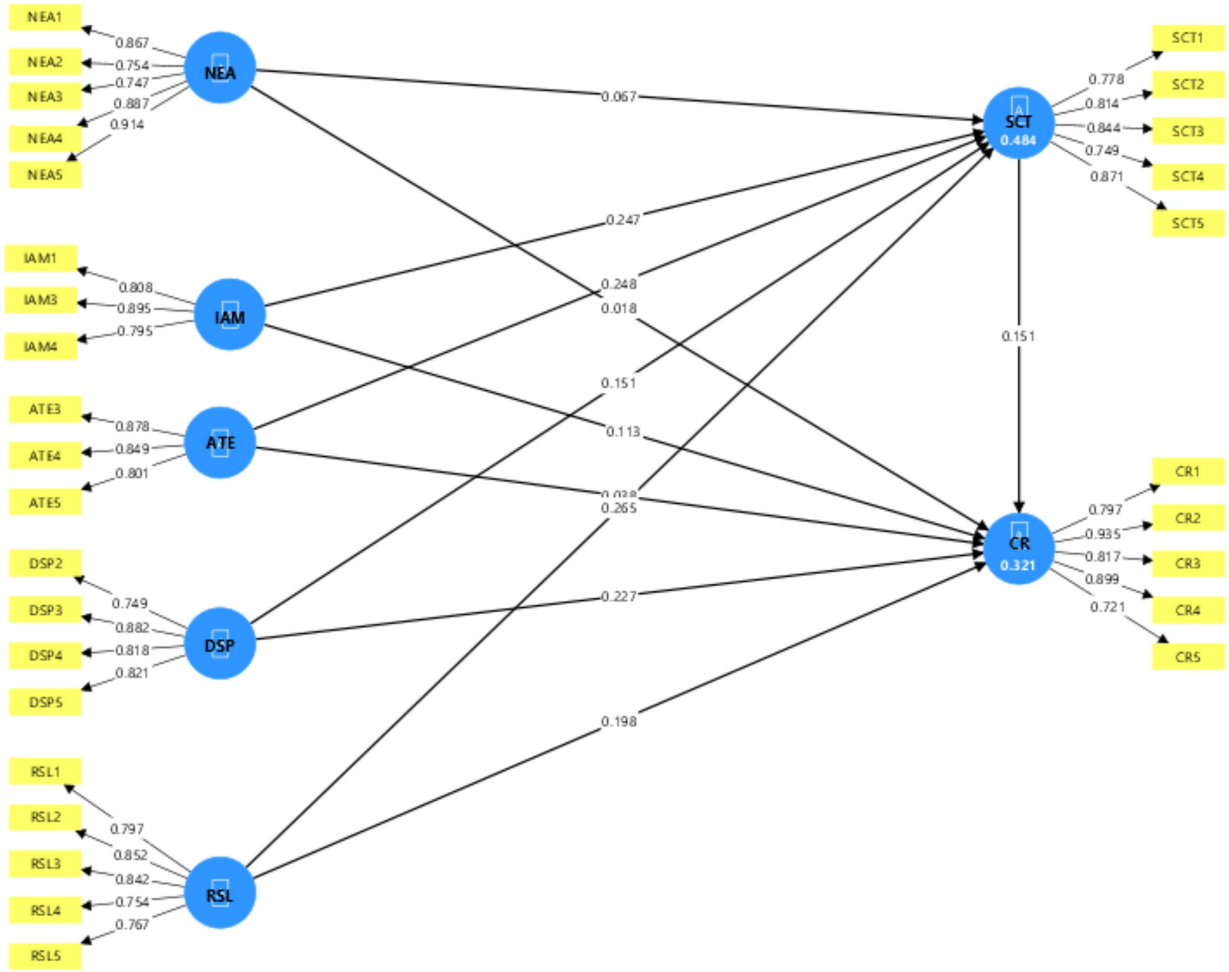
PLS-SEM model structural model including direct and indirect paths for mediation analysis.

SPSS software was used to look at the respondents’ profiles, convergent Validity. We used Smart PLS 4 to test (Figure 2) and confirm the hypotheses of the study model. In the past, covariance-based structural equation modeling was the best way to examine at complicated interactions between observable and latent variables. But in the last few years, PLS-SEM has become much more popular (Hair et al., 2021).

Convergent validity appears when different measures of the same construct exhibit a positive correlation with one another. Measures of convergent validity include factor loadings and average variance extracted (AVE), which must be greater than 0.5 (Hair et al., 2021). As shown in Table 3, all the constructs displayed satisfactory convergent validity, with loadings and AVE values exceeding 0.5. During the initial assessment, the Institutional Accountability Mechanisms, Algorithmic Transparency & Explainability, Data Security & Privacy Resilience, construct did not meet the convergent validity criterion due to an AVE value below 0.50; therefore, item IAM2, IAM5, ATE1, ATE2, DSP1 was removed to improve convergent validity, after which all constructs satisfied the recommended thresholds. The final aspect is discriminant validity (Table 4), the extent to which a construct is empirically different from other constructs. Following the guidelines of Hair et al. (2021), the bootstrapping of 361 samples was applied.

**Table 4:** Discriminant validity—Fornell-Larcker criterion.

Construct	ATE	CR	DSP	IAM	NEA	RSL	SCT
ATE	0.843						
CR	0.333	0.837					
DSP	0.437	0.469	0.819				
IAM	0.390	0.395	0.481	0.834			
NEA	0.033	0.101	0.130	0.167	0.837		
RSL	0.371	0.440	0.461	0.380	0.054	0.803	
SCT	0.512	0.453	0.510	0.529	0.151	0.525	0.812

The measurement model failed to meet the requirements in the initial analysis due to its low AVE value. Five indicators were deleted before running the measurement model once.

### 4.2 Structural model

Examining the structural model is the second step in evaluating PLS-SEM results (Fornell and Larcker, 1981). The direct paths from the independent variables to Customer Retention were retained to examine partial mediation effects, in line with standard PLS-SEM procedures.

When examining a structural model, it is essential to consider its path coefficients and coefficient of determination (R2) for goodness of fit. The researchers assessed the path coefficients of the structured model in line with Hair et al. (2021) using bootstrapping with 361 samples, a two-tailed test, and a significance level of 0.05, as shown in Table 5. The results demonstrate that apart from IAM2, IAM5, ATE1, ATE2, DSP1 all the hypothesized relationships are observed to be statistically significant. As presented in Table 5, Algorithmic Transparency and Explainability (ATE) has a significant positive effect on Customer Retention (CR) (*β* = 0.037, t = 2.338, *p* = 0.019), indicating that greater transparency and explainability in AI-driven banking systems improve customer retention. Similarly, Data Security and Privacy Resilience (DSP) shows a significant positive influence on Customer Retention (*β* = 0.023, t = 2.071, *p* = 0.038), suggesting that stronger data security and privacy measures enhance customers’ willingness to remain with the bank. In the same way, Institutional Accountability Mechanisms (IAM) have a significant positive impact on Customer Retention (*β* = 0.037, t = 2.422, *p* = 0.015), implying that accountability mechanisms in AI-driven services contribute positively to retaining customers. Further, Regulatory and Social Legitimacy (RSL) also exerts a significant positive effect on Customer Retention (*β* = 0.040, t = 2.255, *p* = 0.024), indicating that compliance with regulations and social expectations strengthens customer retention. However, Normative Ethical Alignment (NEA) does not have a significant effect on Customer Retention (*β* = 0.010, t = 1.302, *p* = 0.193), meaning that ethical alignment alone does not directly influence customer retention in the context of AI-driven banking services.

**Table 5:** Path coefficients.

Path	Original sample (O)	Sample mean (M)	Standard deviation (STDEV)	T statistics (|O/STDEV|)	*p* values
ATE → CR	0.037	0.038	0.016	2.338	0.019
DSP → CR	0.023	0.023	0.011	2.071	0.038
IAM → CR	0.037	0.037	0.015	2.422	0.015
NEA → CR	0.010	0.011	0.008	1.302	0.193
RSL → CR	0.040	0.040	0.018	2.255	0.024

Table 6 presents the mediation analysis results, showing that Sustainable Customer Trust (SCT) significantly mediates the relationship between several ethical AI-driven banking factors and Customer Retention (CR). The indirect effect of Algorithmic Transparency and Explainability (ATE) on Customer Retention through Sustainable Customer Trust is positive and significant (*β* = 0.037, t = 2.338, *p* = 0.019), indicating that transparency and explainability in AI systems improve customer retention by enhancing customer trust. Similarly, Data Security and Privacy Resilience (DSP) has a significant indirect effect on Customer Retention through Sustainable Customer Trust (*β* = 0.023, t = 2.071, *p* = 0.038), suggesting that improved data protection strengthens customer trust, which in turn increases retention. Likewise, Institutional Accountability Mechanisms (IAM) demonstrate a significant positive indirect effect on Customer Retention via Sustainable Customer Trust (*β* = 0.037, t = 2.422, *p* = 0.015), implying that institutional accountability enhances trust and thereby promotes retention. In addition, Regulatory and Social Legitimacy (RSL) also shows a significant indirect influence on Customer Retention through Sustainable Customer Trust (*β* = 0.040, t = 2.255, *p* = 0.024), meaning that compliance with regulations and social expectations improves retention by building trust.

**Table 6:** Mediation analysis and HTMT.

Indirect path	Original sample (O)	Sample mean (M)	Standard deviation (STDEV)	T statistics (|O/STDEV|)	*p* values	Results
ATE → SCT → CR	0.037	0.038	0.016	2.338	0.019	Significant
DSP → SCT → CR	0.023	0.023	0.011	2.071	0.038	Significant
IAM → SCT → CR	0.037	0.037	0.015	2.422	0.015	Significant
NEA → SCT → CR	0.010	0.011	0.008	1.302	0.193	Insignificant
RSL → SCT → CR	0.040	0.040	0.018	2.255	0.024	Significant

Heterotrait-monotrait ratio (HTMT)				
Constructs	Original sample (O)	Sample mean (M)	2.5%	97.5%
CR ↔ ATE	0.354	0.357	0.237	0.487
DSP ↔ ATE	0.532	0.529	0.392	0.650
DSP ↔ CR	0.488	0.486	0.363	0.601
IAM ↔ ATE	0.487	0.487	0.348	0.619
IAM ↔ CR	0.417	0.421	0.309	0.536
IAM ↔ DSP	0.581	0.579	0.458	0.686
NEA ↔ ATE	0.065	0.097	0.056	0.155
NEA ↔ CR	0.090	0.112	0.070	0.169
NEA ↔ DSP	0.115	0.131	0.066	0.229
NEA ↔ IAM	0.139	0.157	0.083	0.254
RSL ↔ ATE	0.451	0.452	0.329	0.570
RSL ↔ CR	0.470	0.469	0.359	0.572
RSL ↔ DSP	0.528	0.524	0.400	0.630
RSL ↔ IAM	0.451	0.450	0.350	0.535
RSL ↔ NEA	0.074	0.100	0.063	0.150
SCT ↔ ATE	0.606	0.604	0.485	0.710
SCT ↔ CR	0.456	0.459	0.355	0.553
SCT ↔ DSP	0.568	0.565	0.449	0.666
SCT ↔ IAM	0.629	0.626	0.524	0.715
SCT ↔ NEA	0.125	0.142	0.077	0.236
SCT ↔ RSL	0.604	0.599	0.472	0.707

However, the indirect effect of Normative Ethical Alignment (NEA) on Customer Retention through Sustainable Customer Trust is not significant (*β* = 0.010, t = 1.302, *p* = 0.193), indicating that ethical alignment alone does not significantly enhance customer retention through trust.

GOF = $\sqrt{\mathrm{AVE}*R^{2}}$ = $\sqrt{0.60\times 0.4025\;}$ $=\sqrt{0.2415}=0.491.$

GOF = $\sqrt{\mathrm{AVE}*R^{2}}$ = $\sqrt{0.60\times 0.393\;}$ $=\sqrt{0.2358}=0.486$

Table 7 presents the R-square values and the Goodness of Fit (GoF) results for the endogenous constructs in the model. The R-square value for Sustainable Customer Trust (SCT) is 0.484, indicating that 48.4% of the variance in Sustainable Customer Trust is explained by the exogenous variables. Similarly, the R-square value for Customer Retention (CR) is 0.321, indicating that 32.1% of the variance in Customer Retention is explained by the predictor variables. According to Hair et al. (2021), these values indicate moderate explanatory power of the structural model, which represents a weak to moderate explanatory power. The GoF value of 0.566 for Sustainable Customer Trust indicates a large model fit, while the GoF value of 0.450 for Customer Retention also indicates a large overall model fit, based on the benchmark values suggested by Wetzels et al. (2009), where 0.10, 0.25, and 0.36 represent small, medium, and large fit, respectively. These findings suggest that the proposed model demonstrates strong explanatory power and substantial predictive relevance, indicating that the ethical AI-related constructs meaningfully contribute to enhancing Sustainable Customer Trust and Customer Retention in AI-driven banking services.

**Table 7:** (A) R-squared test and (B) goodness fit.

Endogenous construct	R -Square	R-square adjusted
**A**		
Customer retention	0.321	0.309
Sustainable customer trust	0.484	0.477
**B**		
Sustainable customer trust	0.660	0.484
Customer retention	0.701	0.321

## 5 Discussion

This study investigates the impact of ethical Artificial Intelligence (AI) practices in Customer Relationship Management (CRM) on customer outcomes by applying the EASTL theoretical framework in the context of private sector banking. The model incorporates five key ethical EASTL Ethical Constructs- Normative Ethical Alignment, Institutional Accountability Mechanisms, Algorithmic Transparency and Explainability, Data Security and Privacy, and Regulatory and Social Legitimacy as predictors of customer behavior. Sustainable Customer Trust is introduced as a mediating construct that explains how these ethical AI practices translate into long-term relationship outcomes. The proposed structural model explains 48.4% of the variance in Sustainable Customer Trust and 32.1% of the variance in Customer Retention, based on their respective R-square values, indicating moderate explanatory power. These findings suggest that ethical AI practices substantially influence the development of trust, which in turn enhances customer retention in AI-driven banking services. Accordingly, both direct effects of ethical AI constructs on customer retention and indirect effects through Sustainable Customer Trust were assessed, enabling a clear distinction between immediate impacts and trust-mediated relational outcomes. The mediation analysis reveals that Algorithmic Transparency and Explainability (ATE) has a significant indirect effect on Customer Retention (CR) through Sustainable Customer Trust (SCT) (*β* = 0.037, t = 2.338, *p* = 0.019), supporting the proposed hypothesis. This indicates that when AI systems in banking are transparent and their decisions are explainable, customers develop higher trust, which in turn enhances their likelihood of continuing relationships with the bank. The direct effect of ATE on CR is also significant (*β* = 0.037, t = 2.338, *p* = 0.019), suggesting that transparency not only builds trust but also independently strengthens customer retention. Similarly, Data Security and Privacy (DSP) demonstrate a significant indirect effect on CR via SCT (*β* = 0.023, t = 2.071, *p* = 0.038), confirming its mediating relationship. This implies that robust data protection practices foster customer trust, which subsequently improves retention. The direct effect of DSP on CR is also significant (*β* = 0.023, t = 2.071, *p* = 0.038), indicating that data security plays both a direct and trust-mediated role in retaining customers. The results further show that Institutional Accountability Mechanisms (IAM) significantly influence CR through SCT (*β* = 0.037, t = 2.422, *p* = 0.015), supporting the mediation hypothesis. This suggests that when banks demonstrate accountability in their AI-driven decisions, customers perceive greater reliability and fairness, leading to stronger trust and continued engagement. Additionally, the direct effect of IAM on CR is significant (*β* = 0.037, t = 2.422, *p* = 0.015), highlighting its dual impact on retention. In contrast, Normative Ethical Alignment (NEA) does not exhibit a significant indirect effect on CR through SCT (*β* = 0.010, t = 1.302, *p* = 0.193), indicating that its influence on customer retention is limited in the presence of the mediator. The direct effect of NEA on CR is also insignificant (*β* = 0.010, t = 1.302, *p* = 0.193), suggesting that ethical alignment alone may not be strongly perceived by customers in AI-enabled banking contexts or may operate through other unexamined mechanisms. Finally, Regulatory and Social Legitimacy (RSL) shows a significant indirect effect on CR via SCT (*β* = 0.040, t = 2.255, *p* = 0.024), supporting the mediation hypothesis. This indicates that compliance with regulations and alignment with societal expectations enhance customer trust, which subsequently improves retention. The direct effect of RSL on CR is also significant (*β* = 0.040, t = 2.255, *p* = 0.024), confirming its independent contribution to customer retention. Overall, the findings demonstrate that Sustainable Customer Trust plays a crucial mediating role in translating ethical AI practices into customer retention outcomes. While most constructs (ATE, DSP, IAM, and RSL) exhibit both significant direct and indirect effects—indicating partial mediation, NEA shows no significant mediation effect, highlighting the need for stronger operationalization or customer awareness of ethical alignment in AI-driven banking services.

## 6 Implication

### 6.1 Theoretical implication

This research provides several strong theoretical implications by deploying the EASTL framework (Ethical AI constructs), with corresponding relevance to Customer Relationship Management (CRM) in AI-driven banking, thus complementing the developing literature on ethical artificial intelligence and customer-oriented outcomes. Firstly, this study contributes to theoretical understanding of ethical AI by operationalizing Normative Ethical Alignment, Institutional Accountability Mechanisms, Algorithmic Transparency and Explainability, Data Security and Privacy, and Regulatory and Social Legitimacy as critical antecedents for user-related outcomes. The results corroborate previous trends in arguing that an ethical lens is pivotal in AI implementation within service-ecosystems and that this viewpoint is a critical determinant of customer views and behavior (Mullangi et al., 2018; Ni, 2024). Such an approach of empirically validating these constructs within CRM also brings the ethical AI literature from theoretical to tangible, result driven constructs.

Secondly, this study serves to advance relationship marketing/trust theory by identifying Sustainable Customer Trust (SCT) as an important mediating variable between ethical AI practices and Customer Retention (CR). The strong indirect effect size found for most constructs (ATE, DSP, IAM, and RSL), supports our belief that trust is a fundamental relational asset in the context of AI mediated environments. This supports existing studies stating that trust is built based on the dependability, transparency and data protection of a system in digital service settings (Ni, 2024), and adds to it by showing how ethical AI governance frameworks build systematic long-term trust. The study contributes to increasing the theoretical framework of trust as an outcome, not just an end point, but as the dynamic mediating construct, through which ethical practices become consistent with customer relationships over time.

Third, the study integrates lessons from ethical governance theory and technology-enabled CRM, and suggests that institutional and technological factors interact to effect customer retention outcomes. Other constructs like algorithmic transparency or data security are technological characteristics, but institutional accountability and regulatory legitimacy are organizational rules, that is governance mechanisms, which influence consumer perceptions of fairness and trust. Conceptually, this two way perspective builds on what other research has already suggested by demonstrating that customer behavior inside companies providing AI-enhanced services is not only affected by the efficiency of technology, but also by the customers attitude toward the ethical integrity of the system (Grewal et al., 2020; Wang et al., 2022). The seemingly trivial effect of Normative Ethical Alignment also provides additional guidance to this theory and indicates that abstract ethical principles in theory may provide little visibility and impact, or have a limited direct impact, unless they are implemented as features or practices of the system. Finally, this study supplements the ongoing literature by offering a hybrid framework that combines ethical AI constructs with relationship marketing outcomes, extending traditional CRM models into the context of intelligent systems. The results draw attention to how ethical AI is more than just a compliance issue, it is also an important strategic driver of customer trust and customer retention. With moderate explanatory power (R^2^ values), and excellent model associations, the study supports the study on implementing ethical, technological and relational perspectives in examining customer behavior in AI-embedded banking services. All in all, the theory of this study has broadened, since ethical AI has been posited here as a multi-layered concept with direct and indirect effects on customer outcomes, further underpinned by the fundamental role of trust in mediating the effects. It provides insights for future research to consider other moderating factors, cross-cultural validation and the changing role of ethical AI in developing long-term customer relationships in digital financial ecosystems.

### 6.2 Practical implication

The results may then be useful to bank managers, artificial intelligence (AI) developers, policymakers and CRM strategists for building customer trust and retention regarding AI-driven banking services. First, the results highlight the significance of algorithmic Transparency and Explainability (ATE). Since ATE shows both direct and trust-mediated impacts on customer retention, banks need to ensure that AI-driven decisions — including credit scoring, loan approvals and personalized recommendations — are transparently explained to customers. Understanding and clear explanations, audit trails, easy-to-understand interfaces, and open communication reduce perceived uncertainty and build trust, ultimately improving long-term customer relationships. The second important observation of the research is the significance of DSP in customer behavior. There is, therefore, a need for companies to invest in the robust security of their cybersecurity systems, data encryption, and data usage protocols to encourage transparency in using them. Transparency of the collection and storage as well as protection of customer information can help build trust and reduce perceived risk. And privacy dashboards, and any kind of consent management system, can have customers feel that they really control their personal data, which can improve retention results.

Third, the results highlight the role of institutional accountability mechanisms (IAM). Clear governance measures by banks: AI ethics committees, grievance redressal mechanisms and algorithmic decision accountability. And when customers feel that institutions are both responsible and answerable for their AI outcomes, their faith in the systems does go up. Some practical mechanisms could strengthen accountability and inspire trust over time, such as dispute resolution systems, human supervision when making essential decisions and open process for dealing with complaints. Fourth, the study supports the significance of regulatory & social legitimacy (RSL) on trust and retention. Banks need to comply with regulatory frameworks (which apply to a wide range of industries) and realign their AI practices with societal mandates. Institutional credibility can be strengthened by communicating adherence to data protection regulations and ethical AI guidelines. By creating consistent standards for ethical AI practices in banking and financial services, policymakers can set the stage for a transparent digital ecosystem. On the other hand, the small influence of NEA indicates that abstract ethical principles are unlikely to have a significant impact on customer behavior, unless there is a clear and concrete link between normative ethical orientation and customer behavior. Hence, banks should actively implement ethical values through concrete initiatives such as policy transparency, customer education schemes and ethical AI certification, as opposed to only by expressing ethical beliefs. Last but certainly not least, the study offers managerial insights for digital transformation in banking CRM. By integrating AI with strategies for customer relations in a moral way, banks can guarantee better quality customer service. This will reduce them turning over, and create long-term loyal customers. Explainable AI systems, secure data infrastructures, and governance mechanisms of AI systems are one way to establish a competitive edge.

## 7 Conclusion

The adoption of Artificial Intelligence (AI) in banking has rapidly transformed customer relationship management (CRM) as a field. Hence ethical considerations are essential if customer trust and longevity are to be managed in future. Though other studies have looked at AI adoption or trust entirely in isolation, few have investigated how all ethical AI practices together influence customer retention via systems of trust. In order to fill these gaps, the present study examines the influence of five key EASTL Ethical Constructs -Normative Ethical Alignment, Institutional Accountability Mechanisms, Algorithmic Transparency and Explainability, Data Security and Privacy, and Regulatory and Social Legitimacy on Customer Retention (CR), with Sustainable Customer Trust (SCT) as a mediating variable. The results showed that Sustainable Customer Trust emerges as a key mediator of the adoption of ethical AI practices resulting in customer retention outcomes. Customer retention reflected direct and indirect effect of constructs such as Algorithmic Transparency and Explainability, Data Security and Privacy, Institutional Accountability Mechanisms, and Regulatory and Social Legitimacy, all of which build trust and establish long-term relationships with customers. Specifically, trust in AI-enabled banking services has been found to be greatly driven by transparency and data protection. In contrast, Normative Ethical Alignment was not clearly correlated to customer behavior; otherwise, abstract ethical principles cannot fundamentally affect customer behavior—unless these principles are enacted through observable and actionable behaviors. As a result, the study verifies that ethical AI is not just a compliance issue which requires compliance, but the strategic deciding factor for customer trust and retention. Integrating ethical governance into AI CRM means banks can increase customer confidence, decrease uncertainty, and nurture loyalty among their customer base in a digital-centric financial environment.

### 7.1 Limitation and future study scope

Although the study provides valuable implications for the utilization of the EASTL framework in AI-based CRM, there are limitations. First, the study is limited to private sector banks and a specific geographic context, which may restrict the generalization or application to other banking categories, FinTech platforms, or international settings. Second, it is a cross-sectional study relying on self-reports of the actual user with potential response bias and will not cover the dynamic change of customer trust over time. Third, even if the EASTL constructs were well operationalized, the insignificant impact of Normative Ethical Alignment indicates that customers may not fully understand some ethical dimensions or that the ways they perceive them need to be better measured. Moreover, it shows moderate variance in Sustainable Customer Trust and Customer Retention, suggesting some other influencing factor is present that was not explained by the model. In addition, the study is limited in external validity, which can be further expanded by applying the EASTL framework to a wider range of financial ecosystems and cultures. Longitudinal or experimental designs could provide more insight on causal relationships and emergent trust dynamics. Additional studies could include moderating variables such as digital literacy or perceived risk and new AI governance mechanisms to better understand the effects of ethical AI on CRM outcomes.

## Data Availability

The raw data supporting the conclusions of this article will be made available by the authors, without undue reservation.
